# EHMTI-0041. ARTe Cluster project. Cluster headache - from pain to inspiration

**DOI:** 10.1186/1129-2377-15-S1-C55

**Published:** 2014-09-18

**Authors:** P Rossi, C Geraci, F Mesa Navarro

**Affiliations:** 1 Headache Centre, INI, Grottaferrata (Rome), Italy; 2 Europa, AlCe Cluster, Siegen, Germany

## Background and aim

Barriers of communication play a crucial role in underestimating the disability induced by the headache disorders. Art is at its essence “a tool for creating empathy among human beings”. ARTe Cluster is a project of Alleanza Cefalalgici Cluster aimed to collect artistic material to be used for promoting the activities of the association and to raise awareness about CH patients sufferings. In this study we analyzed the educational, artistic and therapeutic value of the paintings included in ARTe Cluster.

## Methods

In 2011 we solicited CH patients’ associations and figurative artists, throughout the world, to join to the ARTe Cluster project. Here we present a narrative analysis of artwork collected in the last two years.

## Results

ARTe Cluster Project includes now 210 paintings (95 made by Cluster Headache sufferers; http://alcecluster.cefalea.it/). The exhibition expresses the full range of physical and psychological aspects related to CH with a prevalence of themes dealing with exhaustion, torture, isolation and fear.

## Conclusion

CH represents a powerful source of inspiration for artistic expression. Art has proven to be able to create powerful icons of the disease that communicate about CH much more than single words can do and that may be used as educational tools to sensitize people and physicians about the dramatic sufferings of CH patients. An impressive number of CH patients demonstrated an artistic talent (12 of them were professional artists) possibly for the therapeutic aspect of the creative process of art making or for a natural artistic talent.

No conflict of interest.

